# Reliability of Various Measurement Stations for Determining Plantar Fascia Thickness and Echogenicity

**DOI:** 10.3390/diagnostics6020015

**Published:** 2016-04-13

**Authors:** Adebisi Bisi-Balogun, Michael Cassel, Frank Mayer

**Affiliations:** 1 Clinical Exercise Science, University of Potsdam, Am Neues Palais, Potsdam, Brandenburg 14467, Germany; 2 Sports and Health Sciences, University Outpatient Clinic, Am Neues Palais, Potsdam, Brandenburg 14467, Germany

**Keywords:** plantar fascia, reliability, sonography, musculoskeletal

## Abstract

This study aimed to determine the relative and absolute reliability of ultrasound (US) measurements of the thickness and echogenicity of the plantar fascia (PF) at different measurement stations along its length using a standardized protocol. Twelve healthy subjects (24 feet) were enrolled. The PF was imaged in the longitudinal plane. Subjects were assessed twice to evaluate the intra-rater reliability. A quantitative evaluation of the thickness and echogenicity of the plantar fascia was performed using Image J, a digital image analysis and viewer software. A sonography evaluation of the thickness and echogenicity of the PF showed a high relative reliability with an Intra class correlation coefficient of ≥0.88 at all measurement stations. However, the measurement stations for both the PF thickness and echogenicity which showed the highest intraclass correlation coefficient (ICCs) did not have the highest absolute reliability. Compared to other measurement stations, measuring the PF thickness at 3 cm distal and the echogenicity at a region of interest 1 cm to 2 cm distal from its insertion at the medial calcaneal tubercle showed the highest absolute reliability with the least systematic bias and random error. Also, the reliability was higher using a mean of three measurements compared to one measurement. To reduce discrepancies in the interpretation of the thickness and echogenicity measurements of the PF, the absolute reliability of the different measurement stations should be considered in clinical practice and research rather than the relative reliability with the ICC.

## 1. Introduction

Ultrasound imaging (US) as a non-invasive method for examining the plantar fascia is well established in the literature [1,2]. US is a significantly valuable tool in clinical practice and research for evaluating the integrity of soft tissues including those of the foot [3,4], with healthy tendons known to have a well-organized, uniform, hyperechoic pattern of collagen [5,6,7]. While sonography examination of the structural properties of the plantar fascia (PF) is real-time and operator-dependent, several factors including the transducer placement and handling, machine settings and subject positioning may influence size and appearance of the PF [8,9,10]. Two distinct observable characteristics in plantar fasciopathy seen on the sonograph is a gross thickening and hypoechogenicity of the PF at its insertion at the calcaneal tuberosity [6,7,10]. Thus, a reliable quantitative sonography analysis of PF echogenicity is necessary as subjective evaluations are unreliable with poor agreement between sonographers [11].

The goal during rehabilitation for plantar fasciitis and other PF pathologies is to reduce the intensity of heel pain and, consequently, a reduction in the thickening of the PF [8,11]. Since there is a requirement to examine patients at several time points along the rehabilitation process to determine the effectiveness of interventions and examine changes in the structural properties of the PF with the US, it is essential to have a standardized protocol for ultrasound examinations of the PF that is reliable and precise. This will allow the examiner or researcher to know if the differences observed in thickness or echogenicity of the PF along the rehabilitation process are clinically significant or differences arising due to measurement error of US examinations protocols.

However, due to a lack of a standardization, several protocols exist for subject positioning, transducer handling and placement, as well as measurement stations on the sonograph for the quantification of plantar fascia thickness and echogenicity. In a systematic review of up to 34 studies by Mohseni-Bandpei *et al.* [12], which examined the effectiveness of interventions in the management of plantar fasciitis using sonography, the most common measurement stations used to determine PF thickness varied widely from its insertion at the calcaneal tubercle, namely 0.5, 1, 2 and 3 cm distal from its insertion at the calcaneal tubercle. US measurements of PF thickness at its calcaneal insertion, middle region and distal region at the metatarsal heads [13], as well as echogenicity measurements at its medial calcaneal insertion, have been reported to be reliable [11]. However, the reliability of US measurements of PF thickness and echogenicity at the other measurement stations used for US examinations of the PF and for diagnosis of plantar fasciopathy remain unknown. 

The aim of the study was to determine the random error and systematic bias associated with US measurement of PF thickness and echogenicity taken by a single examiner in a test-retest measurement procedure, using a standardized and simple protocol, at its insertion at the medial calcaneal tubercle extending up to 3 cm distally along its length. We hypothesized that relative and absolute reliability would be higher if the mean of three measurements were used rather than two measurements or one measurement. Overall, we expected that through a standardized protocol, PF thickness and echogenicity measurements using three measurements will be reliable with an intraclass correlation coefficient (ICC) of ≥0.75; Standard error of measurement percentage (SEM%) ≤10%; Minimum detectable change (95% CI); and Limits of agreement percentage ≤20%.

## 2. Materials and Methods

### 2.1. Participant Selection

Twelve healthy volunteers (eight males and four females), aged 18 to 31 years, without symptoms of lower extremity disorders, were recruited for this study. Exclusion criteria were prior history of surgery to the foot and current or prior pain in the PF. Participants provided informed consent on forms approved by the ethics committee of the University of Potsdam, Brandenburg.

### 2.2. Test Procedure

**Figure 1:**
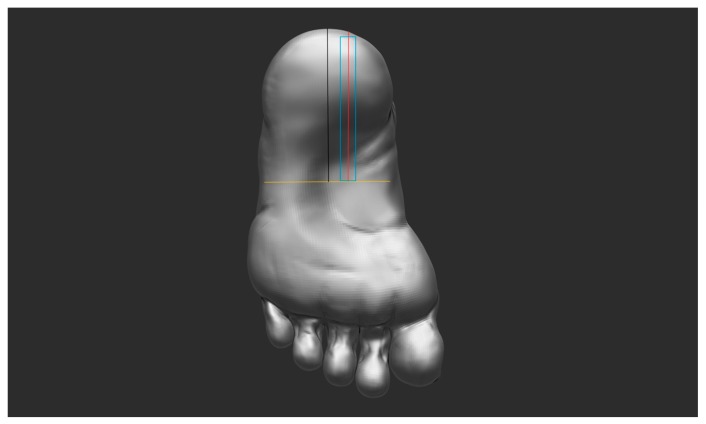
Image showing protocol for standardization of transducer head placement with yellow line marking the width of the sole of the foot at a point aligned parallel with the navicular tuberosity; black line marking the midpoint of the sole of the foot; red line marking placement of middle of transducer probe 1 cm medial to the middle of the foot; the blue outline shows the total surface area of the plantar surface of foot in contact with transducer head.

The scanner used was a Toshiba Xario Prime scanner (Toshiba Medical Systems Corporation, Japan) equipped with an 8 MHz linear transducer probe, with a contact surface area approximately 7 cm in length and 1 cm in width. The settings of the US scanner were kept constant during all measurements to avoid potential changes in the images. All subjects were positioned in supine position and the width of the plantar surface of the foot parallel to the navicular tuberosity was measured. At the identified midpoint of the plantar surface of the foot at this point, a 1 cm line was drawn medial to the identified midpoint and drawn along the plantar surface of the foot, extending to the heel (Figure 1). Subjects were then positioned in a prone position with the toes dorsally flexed, and the talocrural joint was positioned in 0 degree flexion. As the PF is attached at the plantar surface at the toes, dorsal flexion of the toes creates tension in the PF and makes the borders of the fascia more clearly defined. The center of transducer head was aligned parallel to a line 1 cm medial to the midline of the foot to examine the central bundle of the plantar fascia at the insertion onto the medial calcaneus tubercle (Figure 1). A total of three long-axis scans were obtained, with the PF superior and inferior borders, well defined by sonography, where it inserts into the medial calcaneal tubercle identified by a white hyperechoic line. Also, care was taken not to apply pressure to the foot through the handling of the transducer head. Three sonographs of each foot were taken during the test and retest to avoid error due to transducer obliquity, with the transducer head lifted off the surface of the foot and then repositioned for each measurement. Uniform echogenicity of the regions between the superior and inferior borders of plantar fascia was considered a normal finding. The retest was performed approximately 45 min after the first test. During the retest, three additional sonograms of the PF were recorded for each foot. Care was taken to maintain the same standardized foot position, to keep the ultrasound scanner’s settings constant, and to replicate the same measurement. The examiner was highly experienced with use of ultrasound for examination of musculoskeletal soft tissues.

### 2.3. Image Analysis

**Figure 2:**
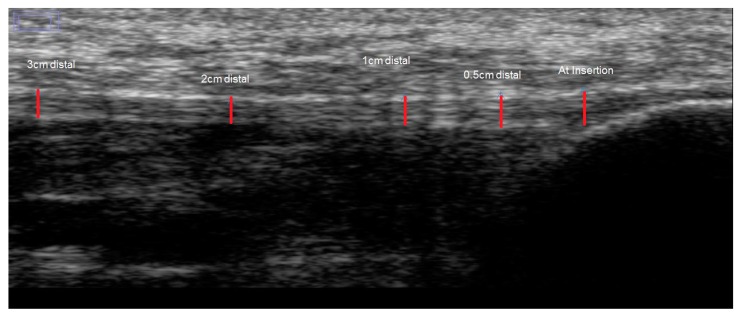
Sonograph showing measurement of plantar fascia (PF) thickness at five various points: its insertion at medial calcaneal tubercle; 0.5, 1, 2 and 3 cm distal, with red lines showing caliper positioning for analysis and measurements.

**Figure 3:**
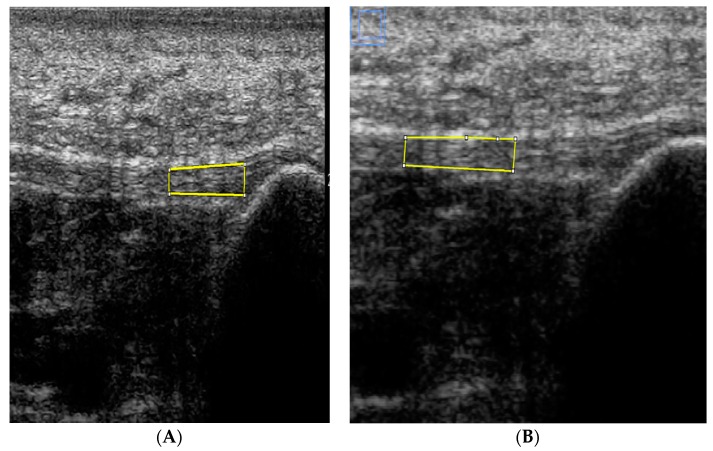
(**A**) Sonograph showing placement of regions of interest (ROI) at measurement stations along the length of the PF distally from insertion (0) to 1 cm distal; and (**B**) 1 to 2 cm distal from its insertion at the medial calcaneal tubercle with the yellow frame indicating the selected ROIs along the lengths of the PF.

All saved sonographs were stored and archived using subject IDs and retrieved in their original formats for subsequent analysis performed by the examiner, blinded to participant information. On each sonograph, the PF thickness and echogenicity was measured from still images of the PF using Image J (National Institute of Health, Bethesda, Rockville, MD, USA), an Image processing and analysis software program (version 1.49), using a zoom of 300% and a calibrated scale of 12.7152 pixels/millimeter. The thickness of the PF (in millimeters) was measured at its medial calcaneal tubercle insertion, and 0.5, 1, 2 and 3 cm distal from its insertion from the medial calcaneal tuberosity. The calibrated digital line was placed aligned parallel with the superior and inferior echoic borders of the fascia at its most visible expansion on the sonograph at each measurement station (Figure 2). Echogenicity was determined by measuring the mean grey level within the selected regions of interest (ROI). It is based on the grey-scale image which for each pixel ranges between 0 (black) and 255 (white), with connective tissues having a higher echogenicity compared to the surrounding muscles [14]. The sum of echogenicity of pixels within the selected ROI is divided by the sum of the pixels within the ROI to derive a mean grey level. The ROI had a constant length of 1 cm and an area ranging between 0.1 and 0.2 cm^2^, covering as much area as possible between the superior and inferior borders of the PF at each of the measurement stations. The area of the ROI varied between 0.1 and 0.2 cm^2^, as although the length of the ROI was fixed at 1 cm, the height had to be adjusted progressively distally due a corresponding decrease in the PF thickness progressively distally (Figure 3A,B). The mean grey level was measured to reduce variance. Also, a mean of the grey level of three sonographs at each of the measurement stations was used as the final outcome (Figure 3A,B).

### 2.4. Statistical Analysis

Statistical analysis was performed using IBM SPSS statistics (IBM, New York, NY, USA) version 21.0. Each sequential measurement of PF was treated in SPSS as a separate variable. The means of the right and left foot for all 12 subjects, obtained using a paired samples *t* test, found no significant differences. Thus, a total of 24 feet (12 subjects) were included in the final analysis. The reliability of the PF thickness and echogenicity was determined using a mean of three measurements where a higher reliability would be expected compared to one single measurement [13,15,16]. Also, a mean of three measurements from three different sonographs, as opposed to three measurements from one sonograph, was used to take into account random errors and systematic bias which may be attributed to each scan. Rathcleff *et al.* [16] reported that the scan itself may be a contributing source of error in US examinations of the PF other than errors in measurements of the scan alone between the test and retest. The relative reliability was determined using the intraclass correlation coefficient (ICC 95% CI), while absolute reliability was determined using the limits of agreement (LOA); Standard Error of Measurement (SEM); minimum detectable change (MDC_95_); and smallest real difference (SRD) [17,18,19]. Where:

(1) SEM=SD√(1−ICC) and SEM%=(SEM/mean)×100

(2) $${\text{MDC}}_{95}=\text{SEM}\times 1.96\times \sqrt{2}\text{ and }{\text{MDC}}_{95}\%=({\text{MDC}}_{95}/\text{mean})\times 100$$

(3) $$\text{SRD}=1.96\sqrt{\;}\left(2\times \text{SEM}\right)$$

LOA = BIAS ± 1.96SD and LOA% = (Bias/mean) × 100
(4)

## 3. Results

The results of the US measurements of both the thickness and echogenicity of the PF are summarized in Table 1 using means and standard deviations. The results show a progressive decrease in the thickness and echogenicity of the PF along its length as it extends distally from its insertion at the calcaneal tubercle.

### 3.1. Relative Reliability

The ICC coefficients for each of the five measurement stations for thickness are summarized in Table 2. Four out of the five measurement stations showed excellent reliability of the thickness of the PF measurement (ICC > 0.90), while good reliability was shown at 0.5 cm distally (ICC > 0.75). In addition, all three measurement stations for echogenicity showed excellent reliability (ICC > 0.90) (Table 2).

### 3.2. Absolute Reliability

#### 3.2.1. PF Thickness

At all five measurement stations, the SEM ranged from 0.08–0.15 mm with a low SEM% obtained between 3.4%–5.2% (SEM% ≤ 10%) (Table 2). A comparison of the five measurement stations revealed the most absolute reliable measurement station with the lowest systematic bias and random error was at the measurement of the PF thickness at 3 cm distal from its insertion at the medial calcaneal tubercle, showing the lowest values for LOA, SEM, MDC_95_ and SRD. The least absolute reliable measurement station with the highest random error and systematic bias was at 0.5 cm distal from its insertion at the medial calcaneal tubercle, showing the highest values for LOA, SEM, MDC_95_ and SRD out of all five measurement stations (Table 2).

#### 3.2.2. PF Echogenicity

At all three measurement stations, the SEM of the mean grey scale level ranged from 5.2–6.6 with a relatively low SEM% between 6.3%–7.0% (Table 2). At all three measurement stations, a SEM% ≤ 10% was obtained. A comparison of the three measurement stations revealed that the most absolute reliable measurement station for PF echogenicity, and which had the lowest systematic bias and random error, was at the measurement of the PF echogenicity at an ROI extending from 1 cm distal to 2 cm distal from its insertion at the medial calcaneal tubercle, showing the lowest values for LOA, SEM, MDC_95_ and SRD. The least absolute reliable measurement station with the highest systematic bias and random error was at the measurement station with a ROI extending from its insertion at the medial calcaneal tubercle to 1 cm distal, showing the highest values for LOA, SEM, MDC_95_ and SRD values (Table 2).

## 4. Discussion

In the present study, we investigated the relative and absolute reliability of various measurement stations for measuring the thickness and echogenicity of the PF using sonography. In our sample of a healthy population of students that were recreationally active with no lower limb and specifically no PF pathology, the relative reliability (ICC) was good to excellent for all five measurement stations for PF thickness, and excellent for all three measurement stations for echogenicity. Using a mean of three measurements from three sonographs, compared with one measurement from one sonograph, showed a higher relative and absolute reliability with reduced systematic bias and random error. However, the results showed a good-to-excellent reliability for thickness and an excellent reliability for echogenicity measurements using one sonograph. While using the ICC as an estimate of reliability is common amongst reliability studies, the ICC is not without its limitations. In this study, the measurement stations with the highest ICCs for PF thickness and echogenicity did not necessarily have the least systematic bias and random error. 

### 4.1. PF Thickness

Using a mean of three measurements from three sonographs, the ICCs for all the measurement stations of thickness of the PF was higher than those reported in previous studies with an ICC ≥ 0.88 at the five measurement stations examined in this study. The highest intra-rater ICC was observed 1 cm distal from the insertion at the medial calcaneal tubercle. In their studies, Rathcleff *et al.* [16] and Cheng *et al.* [11], respectively, reported an intra-rater ICC of 0.67 and 0.77 [15] and an intra-rater ICC of 0.86 and 0.87 [11] for the two examiners in their respective studies using a measurement station at the insertion of the PF at the calcaneal tubercle. 

For the determination of absolute reliability, the measurement station at 3 cm distal from the insertion of the PF at the medial calcaneal tubercle showed the lowest SEM, MDC_95_, SRD and LOA compared to the other measurement stations. The LOA and LOA% reported in this study for all measurement stations was lower than that reported by Rathcleff *et al.* [16], who reported a LOA of 0.8 and 0.9 mm, respectively, and a LOA% of 21% and 25%, respectively, for the intra-rater reliability of the two examiners in their study, using the insertion of the PF at the calcaneal tubercle as their measurement station for PF thickness measurement.

### 4.2. Echogenicity of PF

Using the means of three measurements, the intra-rater ICCs for all the measurement stations (selected ROI) of PF echogenicity examined in this study were similar to those reported by Cheng *et al.* [11]. In their study, they reported an ICC of 0.92 and 0.93 for their two examiners using a measurement station with a ROI extending from the insertion of the PF at the medial calcaneal tubercle, with an area between 0.1 cm^2^ to 0.2 cm^2^. Using a similar ROI, the results of this study showed an ICC of 0.95.

In comparing the methodology of this study to that employed by Cheng *et al.* [11] and Rathcleff *et al*. [16], the positioning of the transducer head was standardized and clearly defined in this study and was highly repeatable and reproducible between measurements. Additionally, the retest measurements in this study was performed 45 min after the first examination, whereas in the study by Cheng *et al.* [11], the retest was performed a week after the first measurements. Several factors including physical or recreational activities performed by the subject within this period may have resulted in changes in the structural properties of the PF between the test and retest [20].

The results of this study showed a decrease in thickness and echogenicity of the PF from its insertion at the medial calcaneal tubercle to 3 cm distally. From the results of this study, we cannot make a comparison of PF echogenicity patterns at different points along its entire length as the length of the central bundle of the PF between its attachments at the metatarsal heads and medial calcaneal tubercle ranges between 125 ± 2.0 mm [21] and 146 ± 9.6 mm [22], far beyond the ROIs under consideration in this study. However, the decrease in echogenicity observed distally up to 3 cm is not contrary to what may be expected. During stance, the maximum stress on the PF averages about 96% ± 36% of body weight, with the PF transmitting these large forces between the hind foot and forefoot during stance, as well as from the Achilles tendon to the forefoot in the latter part of the stance phase of gait [23]. At heel strike, PF stress is at its lowest compared with the other phases of the gait cycle and also concentrated at the medial calcaneal tubercle [24]. This stress gradually increases during mid-stance and peaks at push off [23,24], rising up to five times the initial value seen at heel strike [24]. Additionally, the stress generated in the PF during midstance and push off is highest at the distal region of the PF at its attachment at the metatarsal heads, followed by its ends at the medial calcaneal insertion, and lowest in the midportion of the fascia. During gait, there is a concentration of PF stress at the medial calcaneal tubercle and this is related with its being a frequent location of heel pain in plantar fasciopathy [24].

Studies of Achilles and patella tendons have reported increased type 1 collagen and protein synthesis following both acute [25,26,27] and repetitive loading [28,29,30] with the increase in collagen expression and proteins synthesis thought to be regulated by the strain induced in the fibroblasts, facilitating up to a 3 fold increase in collagen formation [25,26,27,28,29,30]. The PF exhibits a non-linear stress-strain curve [31] and therefore, it may be hypothesised that regions of PF with a higher stress distribution when loaded and consequently, strain induced in its fibroblasts, may have a higher echogenicity compared with regions with much lower PF stress distribution during loading. It may be plausible to expect that PF echogenicity at its regions at its distal ends at its insertions at the metatarsal heads and at the medial calcaneal tubercle are higher than the middle portion of the PF. Also, PF echogenicity may also be expected to be highest at its distal region at its attachments at the metatarsal heads. This may explain the decrease in echogenicity observed distally from its medial calcaneal insertion up to 3 cm distally towards the mid portion of the PF from the results of this study. McNally *et al*. [10] reported that plantar fasciitis occur mostly at the PF calcaneal insertion, traumatic injuries at 2 to 3 cm distal from the PF insertion at the calcaneal tubercle and PF fibromatosis, along the distal two thirds of the length of the PF. It is unclear if there is an association between changes in structural properties of the PF and higher risk or incidences of specific pathologies along its structure. Also, craniocaudal echogenicity patterns of the PF along its structure should be further investigated in future studies.

### 4.3. Strengths and Limitations

This study was able to reproduce the placement of the transducer head on the plantar surface of the feet for US examinations of the PF between the test and retest. Also, the result of absolute reliability variables were considered as a more far-reaching indication of reliability than the ICC. Understanding the absolute reliability of outcome variables obtainable with sonography examination of the PF is important for both clinical and research purposes. Knowledge and understanding of the measurement errors associated with each measurement stations would allow the researcher and the clinician to determine if the changes seen or measured between examinations at different time points along the rehabilitation process are clinically significant and due to an effect of the intervention, or as a result of measurement errors [32].

The inter-observer reliability of each of the measurement stations for PF thickness and echogenicity was not determined in this study, although it is expected to be lower than the intra-rater reliability. Also, due to a lack of adjacent muscle for comparing relative mean grey level, the absolute mean grey level was used in the evaluation of echogenicity [11].

## 5. Conclusions

In clinical practice, there is often a necessity to examine the thickness and echogenicity of the PF at different regions along it structure. Differences in absolute and relative reliability of US measurements of PF thickness and its echogenicity exist at different measurement stations, with reliability also determined by the number of measurements taken. The highest absolute reliability was obtained at the measurement station 3 cm distal from its insertion at the medial calcaneal tubercle for PF thickness and, for echogenicity measurement, at a region of interest from 1 to 2 cm distal from its insertion at the medial calcaneal tubercle.

## Figures and Tables

**Table 1:** Showing means and standard deviations of both the test and retest for measurement of plantar thickness and echogenicity and the differences between measurements (ROI = region of interest) using the mean of three measurements from three sonographs.

Measurement Stations	Test (*n* = 24)	Retest (*n* = 24)	Difference (*n* = 24)
	Mean ± SD	Mean ± SD	Mean ± SD
*Thickness (mm)*			
At insertion	3.06 ± 0.6	3.07 ± 0.7	0.17 ± 0.1
0.5 cm distal	3.06 ± 0.5	3.06 ± 0.5	0.25 ± 0.2
1 cm distal	2.83 ± 0.7	2.77 ± 0.7	0.13 ± 0.1
2 cm distal	2.47 ± 0.6	2.42 ± 0.6	0.17 ± 0.1
3 cm distal	2.26 ± 0.4	2.20 ± 0.4	0.13 ± 0.1
*Echogenicity*			
ROI at insertion to 1 cm distal	89.4 ± 29	91.5 ± 30	9.5 ± 10
ROI at 1 to 2 cm distal	81.4 ± 26	83.7 ± 28	7.0 ± 9
ROI at 2 to 3 cm distal	79.2 ± 26	78.7 ± 27	8.3 ± 7

**Table 2:** Showing ICCs, SEMs, SEMs%, MDC_95_ and MDC_95_% for the thickness and echogenicity at all measurement stations using one sonograph and the mean of three sonographs, where ICC = intraclass correlation coefficient; CI = confidence interval; SEM = standard error of measurement, SEM% = relative standard error of measurement; MDC_95_ = minimal detectable change at a 95% confidence level; MDC_95_% = relative minimum detectable change at 95% confidence level; SRD = Smallest Real Difference; and LOA = limits of agreement.

Measurement Stations		ICC	LOA	LOA	SEM	SEM	MDC_95_	MDC_95_	SRD
		(95% CI)	Bias ± 1.96 SD	%		%		%	
Thickness (*mm*)	One sonograph								
	At insertion	0.97 (0.92–0.99)	0.18 ± 0.25	5.6	0.11	3.4	0.30	9.6	0.9
	0.5 cm distal	0.86 (0.63–0.94)	0.30 ± 0.34	9.8	0.17	5.6	0.48	15.6	1.2
	1 cm distal	0.92 (0.78–0.96)	0.22 ± 0.66	7.7	0.19	6.9	0.54	19.1	1.2
	2 cm distal	0.97 (0.92–0.99)	0.16 ± 0.28	6.7	0.10	4.2	0.30	11.6	0.9
	3 cm distal	0.91 (0.76–0.97)	0.17 ± 0.34	7.8	0.12	5.4	0.33	15.1	1.0
	Mean of three sonographs								
	At insertion	0.97 (0.92–0.98)	0.17 ± 0.25	5.6	0.10	3.5	0.29	9.6	0.9
	0.5 cm distal	0.88 (0.70–0.95)	0.25 ± 0.36	8.3	0.15	5.2	0.44	14.3	1.1
	1 cm distal	0.98 (0.96–0.99)	0.13 ± 0.21	4.3	0.09	3.4	0.27	9.3	0.9
	2 cm distal	0.96 (0.90–0.98)	0.16 ± 0.31	6.3	0.10	4.6	0.30	12.8	0.9
	3 cm distal	0.95(0.92–0.96)	0.13 ± 0.20	5.8	0.08	3.7	0.23	10.4	0.8
Echogenicity	One sonograph								
	ROI from insertion to 1 cm distal	0.93 (0.83–0.97)	10.9 ± 21.1	11.6	7.5	8.0	20.9	22.2	7.6
	RO1 from 1 cm to 2 cm distal	0.96 (0.91–0.99)	7.1 ± 15.1	8.3	5.2	6.0	14.3	16.7	6.3
	ROI from 2 cm to 3 cm distal	0.94 (0.86–0.98)	11.3 ± 15.8	13.0	6.5	8.2	17.9	22.7	7.0
	Mean of three sonographs								
	ROI from insertion to 1 cm distal	0.95 (0.85–0.99)	9.5 ± 18.5	10.5	6.3	7.0	17.5	19.4	7.0
	ROI from 1 cm to 2 cm distal	0.96 (0.86–0.97)	7.0 ± 16.6	8.2	5.2	6.3	14.3	17.6	6.3
	ROI from 2 cm to 3 cm distal	0.96 (0.89–0.98)	8.3 ± 13.1	10.5	5.2	6.6	14.4	18.2	6.3

## References

[1] Fornage B.D., Rifkin M.D. (1988). Ultrasound examination of the hand and foot. Radiol. Clin. N. Am..

[2] Cardinal E., Chhem R.K., Beauregard C.G., Aubin B., Pelletier M. (1996). Plantar fasciitis: Sonographic evaluation. Radiology.

[3] Kannus P., Jozsa L. (1991). Histopathological changes preceding spontaneous rupture of a tendon. A controlled study of 891 patients. J. Bone Jt. Surg. Am..

[4] Martinoli C., Bianchi S., Derchi L.E. (1999). Tendon and nerve sonography. Radiol. Clin. N. Am..

[5] Fornage B.D., Rifkin M.D. (1988). Ultrasound examination of tendons. Radiol. Clin. N. Am..

[6] Nuran Sabir M.D., Semra Demirlenk M.D., Baki Yagci M.D., Nevzat Karabulut M.D., Sibel Cubukcu M.D. (2005). Clinical Utility of Sonography in Diagnosing Plantar Fasciitis. J. Ultrasound Med..

[7] McPoil T.G., Martin R.L., Cornwall M.W. (2008). Heel pain–plantar fasciitis: Clinical practice guidelines linked to the international classification offunction, disability, and health from the orthopaedic section of the American Physical Therapy Association. J. Orthop. Sports Phys. Ther..

[8] Liang H.W., Wang T.G., Chen W.S., Hou S.M. (2007). Thinner plantar fascia predicts decreased pain after extracorporeal shock wave therapy. Clin. Orthop. Relat. Res..

[9] Hammer D.S., Adam F., Kreutz A. (2005). Ultrasonographic evaluation at 6-month follow-up of plantar fasciitis after extracorporeal shock wave therapy. Arch. Orthop. Trauma Surg..

[10] McNally E., Shetty S. (2010). Plantar Fascia: Imaging Diagnosis and Guided Treatment. Semin. Musculoskelet. Radiol..

[11] Cheng J., Tsai W., Yu T., Huang K. (2012). Reproducibility of Sonographic Measurement of Thickness and Echogenicity of the Plantar Fascia. J. Clin. Ultrasound.

[12] Mohseni-Bandpei M.A., Nakhaee M., Mousavi M.E., Shakourirad A., Safari M.R., Vahab Kashani R. (2014). Application of ultrasound in the assessment of plantar fascia in patients with plantar fasciitis: A systematic review. Ultrasound Med. Biol..

[13] Crofts G., Angin S., Mickle K., Hill S., Nester C. (2014). Reliability of ultrasound for measurement of selected foot structures. Gait Posture.

[14] Mayans D., Cartwright MS., Walker F.O. (2011). Neuromuscular ultrasonography: Quantifying muscle and nerve measurements. Phys. Med. Rehabil. Clin. N. Am..

[15] Cameron A.F., Rome K., Hing W.A. (2008). Ultrasound evaluation of the abductor hallucis muscle: Reliability study. J. Foot Ankle Res..

[16] Rathleff M., Moelgaard C., Olesen J. (2011). Intra- and Interobserver Reliability of Quantitative Ultrasound Measurement of the Plantar Fascia. J. Clin. Ultrasound.

[17] Atkinson G., Nevill A.M. (1998). Statistical methods for assessing measurement error (reliability) in variables relevant to sports medicine. Sports Med..

[18] Lohr K.N., Aaronson N.K., Alonso J., Burnam M.A., Patrick D.L., Perrin E.B., Roberts J.S. (1996). Evaluating quality-of-life and health status instruments: Development of scientific review criteria. Clin. Ther..

[19] Schwenk M., Gogulla S., Englert S., Czempik A., Hauer K. (2012). Test–retest reliability and minimal detectable change of repeated sit-to-stand analysis using one body fixed sensor in geriatric patients. Physiol. Meas..

[20] Welk A., Haun D., Clark T., Kettner N. (2015). Use of high-resolution ultrasound to measure changes in plantar fascia thickness resulting from tissue creep in runners and walkers. J. Manip. Physiol. Ther..

[21] Gefen A. (2003). The *in vivo* elastic properties of the plantar fascia during the contact phase of walking. Foot Ankle Int..

[22] Chen D.-W., Li B., Aubeeluck A., Yang Y.-F., Huang Y.-G., Zhou J.-Q., Yu G.-R. (2014). Anatomy and biomechanical properties of the plantar aponeurosis: A cadaveric study. PLoS ONE.

[23] Erdemir A., Hamel A.J., Fauth A.R., Piazza S.J., Sharkey N.A. (2004). Dynamic loading of the plantar aponeurosis in walking. J. Bone Jt. Surg. Am..

[24] Gu Y., Li Z. (2012). Mechanical Information of Plantar Fascia during Normal Gait. Phys. Procedia.

[25] Miller B.F., Olesen J.L., Hansen M., Døssing S., Crameri R.M., Welling R.J., Langberg H., Flyvbjerg A., Kjaer M., Babraj J.A. (2005). Coordinated collagen and muscle protein synthesis in human patella tendon and quadriceps muscle after exercise. J. Physiol..

[26] Langberg H., Rosendal L., Kjær M. (2001). Training-induced changes in peritendinous type I collagen turnover determined by microdialysis in humans. J. Physiol..

[27] Kjær M., Langberg H., Heinemeier K., Bayer M., Hansen M., Holm L., Doessing S., Kongsgaard K., Krogsgaard M., Magnusson P. (2009). From mechanical loading to collagen synthesis, structural changes and function in human tendon. Scand. J. Med. Sci. Sports.

[28] Couppe´ C., Kongsgaard M., Aagaard P., Hansen P., Bojsen-Moller J., Kjaer M., Magnusson S.P. (2008). Habitual loading results in tendon hypertrophy and increased stiffness of the human patellar tendon. J. Appl. Physiol..

[29] Kovanen V. (1989). Effects of ageing and physical training on rat skeletal muscle. An experimental study on the properties of collagen, laminin, and fibre types in muscles serving different functions. Acta Physiol. Scand. Suppl..

[30] Magnusson S.P., Langberg H., Kjaer M. (2010). The pathogenesis of tendinopathy: Balancing the response to loading. Nat. Rev. Rheumatol..

[31] Fessel G., Jacob H.A.C., Wyss C.H., Mittlmeier T., Müller-Gerbl M., Büttner A. (2014). Changes in length of the plantar aponeurosis during the stance phase of gait — An * in vivo * dynamic fluoroscopic study. Ann. Anat..

[32] Bisi-Balogun A., Torlak F. (2015). Outcomes following Hip and Quadriceps Strengthening Exercises for Patellofemoral pain syndrome: A Systematic Review and Meta-Analysis. Sports.

